# Post-COVID-19: Time to Change Our Way of Life for a Better Future

**DOI:** 10.3390/epidemiologia5020015

**Published:** 2024-05-22

**Authors:** Roch Listz Maurice

**Affiliations:** Groupe Biomédical Montérégie, Centre Intégré de Santé et des Services Sociaux de la Montérégie-Centre (CISSSMC), Brossard, QC J4W 3J8, Canada; roch-listz.maurice.cisssmc16@ssss.gouv.qc.ca

**Keywords:** COVID-19, pandemics, population density, prevention, public health

## Abstract

**Background and Objectives:** From the year 1 anno Domini until 1855, with the third plague, major pandemics occurred on average every 348 years. Since then, they have occurred on average every 33 years, with coronavirus disease 2019 (COVID-19) now underway. Even though current technologies have greatly improved the way of life of human beings, COVID-19, with more than 700,000,000 cases and 6,950,000 deaths worldwide by the end of 2023, reminds us that much remains to be done. This report looks back at 18 months of COVID-19, from March 2020 to August 2021, with the aim of highlighting potential solutions that could help mitigate the impact of future pandemics. **Materials and Methods:** COVID-19 data, including case and death reports, were extracted daily from the Worldometer platform to build a database for the macroscopic analysis of the spread of the virus around the world. Demographic data were integrated into the COVID-19 database for a better understanding of the spatial spread of the SARS-CoV-2 virus in cities/municipalities. Without loss of generality, only data from the top 30 (out of 200 and above) countries ranked by total number of COVID-19 cases were analyzed. Statistics (regression, *t*-test (*p* < 0.05), correlation, mean ± std, etc.) were carried out with Excel software (Microsoft^®^ Excel^®^ 2013 (15.0.5579.1001)). Spectral analysis, using Matlab software (license number: 227725), was also used to try to better understand the temporal spread of COVID-19. **Results:** This study showed that COVID-19 mainly affects G20 countries and that cities/municipalities with high population density are a powerful activator of the spread of the virus. In addition, spectral analysis highlighted that the very first months of the spread of COVID-19 were the most notable, with a strong expansion of the SARS-CoV-2 virus. On the other hand, the following six months showed a certain level of stability, mainly due to multiple preventive measures such as confinement, the closure of non-essential services, the wearing of masks, distancing of 2 m, etc. **Conclusion:** Given that densely populated cities and municipal areas have largely favored the spread of the SARS-CoV-2 virus, it is believed that such a demographic context is becoming a societal problem that developed countries must address in a manner that is adequate and urgent. COVID-19 has made us understand that it is time to act both preventatively and curatively. With phenomenological evidence suggesting that the next pandemic could occur in less than 50 years, it may be time to launch new societal projects aimed at relieving congestion in densely populated regions.

## 1. Introduction

Since the first known cases identified in Wuhan, China, in December 2019, coronavirus disease 2019 (COVID-19) has rapidly spread throughout the world [1]. Indeed, COVID-19 is a contagious disease caused by the virus SARS-CoV-2, which quickly led to a pandemic.

The symptoms of COVID 19 may include fever [2], cough, headache [3], fatigue, breathing difficulties, loss of smell [4], and loss of taste [5]. In 2020, the Centers for Disease Control and Prevention (CDCP) noted that 14% of COVID-19 patients developed severe symptoms (dyspnea, hypoxia, or lung involvement), while 5% developed critical symptoms (respiratory failure, shock, or multiple organ dysfunction) [6].

Some people continue to experience a range of effects for months or years after infection with the virus, and damage to organs has been observed; this is now called “long COVID” [7]. It has also been observed that older people are at a higher risk of developing severe symptoms.

The transmission of SARS-CoV-2 through direct person-to-person contact has been recognized since the early stages of the COVID-19 pandemic [8]. Although the risk is highest when people are in close proximity, it appears that the virus can be transmitted over longer distances through the inhalation of virus-laden aerosols [9,10]. It should be noted that aerosols are small respiratory particles that can linger in the air and disperse or travel up to 2 m in certain circumstances [11].

There is much more to say about the SARS-2 coronavirus, including variants [12,13,14], virology [15,16], pathophysiology [17,18], diagnosis, prevention [19,20,21,22,23,24,25], treatment [26,27,28,29], mortality, etc. While these topics are well documented elsewhere, they are also well beyond the scope of the present investigation.

This study revisited and retrospectively analyzed 18 months of data on cases and deaths in the early stages of the COVID-19 pandemic. Given the heavy burden of deaths and cases worldwide, the main objective of this study was to highlight potential solutions that could help mitigate the impact of future pandemics. From this perspective, we analyzed the spatial and temporal spreads of the SARS-CoV-2 virus.

## 2. Materials and Methods

From March 2020 to August 2021 inclusive, data related to COVID-19, i.e., the toll of cases and deaths, were extracted daily from the Worldometer platform [30] to constitute a database in Excel format. At the beginning of this study, the toll of cases and deaths was quite coherent with data from the World Health Organization [31]. Worldometer COVID-19 data have been used by many countries and official institutions, including the UK government, the Johns Hopkins CSSE, and the New York Times [32]. Very briefly, Worldometer is a reference site that provides real-time counters and statistics on various topics. It is run by an international team of developers, researchers, and volunteers with the aim of making world statistics available in a thought-provoking and time-relevant format to a wide audience around the world. They claim to be completely independent and self-funded through automated programmatic advertising sold in real time across multiple ad exchanges [32]. While Worldometer data made it possible to carry out a macroscopic analysis of the spread of COVID-19 taking into account the majority of countries in the world, some statistics were also extracted from several government communication sites and from Statista [33] in order to perform a more specific analysis based on the spread of COVID-19 in cities and municipal areas. Furthermore, demographic data (population, area, population density) were extracted from numerous websites, including Wikipedia [34] and La Banque Mondiale [35]. Demographic data were integrated into the COVID-19 database. Without loss of generality, only data from the top 30 (out of 200 and above) countries ranked by total number of COVID-19 cases were analyzed. Statistics (regression, correlation, mean ± std, etc.) were carried out with Excel software. Spectral analysis, using Matlab software, was also used to study the temporal spread of COVID-19.

## 3. Results

Table 1 shows the top 30 countries ranked by the total number of COVID-19 cases as of September 2021. It is observed that the first 11 are exclusively G20 members, while 80% of the first 20 and 73% of the first 30 are G20 members. At that time, those countries accounted for 81% (362,444,064/445,277,485) of the total COVID-19 cases, 83% (4,978,325/6,016,074) of the total number of deaths from COVID-19, as well as 47% (3,783,837,303/8,000,000,000) of the world’s population. It should be noted that G20 includes the European Union, which itself includes 27 countries [34].

It should be noted that a good linear correlation was observed between the number of cases and the respective number of deaths depending on the country, i.e., y = 0.0121x + 19,559 with R^2^ = 0.8042. From that, the following analysis then focused mainly on the number of cases.

### 3.1. Impact of Population Density on the Spread of COVID-19 in Cities and Municipal Areas

Table 2 presents a summary view of the population density of the cities and municipal areas among the most affected in their respective countries by COVID-19. Although the average population density of a country is only about 61 inhabitants per km^2^ [35], it has been observed that some cities and municipal areas have thousands of inhabitants per km^2^. To be more precise, the countries in Table 2 represent a median value of 118 inhabitants per km^2^ with [min; max] = [4; 531] in the year 2021, while cities and municipal areas show a median value of 4100 with [min; max] = [1283; 28,154].

Apart from population density, the ranking of cities (or municipal areas) based on the total number of COVID-19 cases in their respective countries as of September 2021 is shown in Table 2. It has been observed that cities (or municipal areas) with high population densities constitute a powerful activator of the spread of COVID-19. This sample of cities and countries is not exclusive. This list is presented primarily for illustration purposes.

### 3.2. Spectral Analysis of the Temporal Evolution of COVID-19

**Figure 1:**
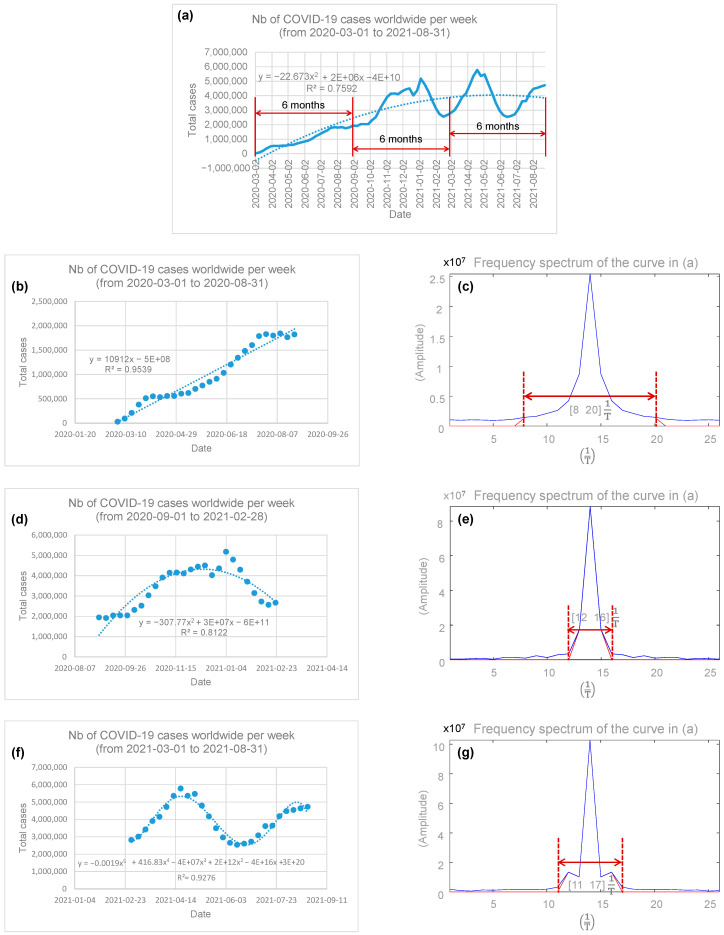
(**a**) The number of COVID-19 cases worldwide by week, from 1 March 2020 to 31 August 2021. The quadratic regression profile (R^2^ = 0.7592) appears to indicate some stability in the spread trend of the SARS-CoV-2 virus towards fall 2020. The data were sampled over three consecutive 6-month periods, i.e., from 2 March 2020 to 31 August 2020, from 1 September 2020 to 28 February 2021, and from 1 March 2021 to 31 August 2021 for further investigations. (**b**) The number of COVID-19 cases worldwide by week, from 1 March 2020 to 31 August 2020; (**c**) COVID-19 spectrum showing width of $\left[8\;20\right]\frac{1}{T}$ at threshold 5% of maximum amplitude, T = 1 week period; (**d**) number of COVID-19 cases worldwide by week, from 1 September 2020 to 28 February 2021; (**e**) spectrum of COVID-19 spread profile showing wide bandwidth $\left[12\;16\right]\frac{1}{T}$, T = 1 week period; (**f**) number of COVID-19 cases worldwide by week, from 1 March 2021 to 31 August 2021; (**g**) spectrum of COVID-19 spread profile showing wide bandwidth $\left[11\;17\right]\frac{1}{T}$, T = 1 week period.

Figure 1a shows the number of COVID-19 cases worldwide by week, from 1 March 2020 to 31 August 2021. For that period, a quadratic regression profile (R^2^ = 0.7592) of the spread of the SARS-CoV-2 virus offered some hope since some stability in the trend could be observed towards fall 2020. Nevertheless, given the complex profile of the COVID-19 spread curve, it was deemed potentially more informative to analyze the data sampled over three consecutive 6-month periods, i.e., from 2 March 2020 to 31 August 2020, from 1 September 2020 to 28 February 2021, and from 1 March 2021 to 31 August 2021; this is shown in Figure 1a. The following sections present the results for each of these periods, separately.

1. COVID-19 spread from 2 March 2020 to 31 August 2020

Figure 1b shows the number of COVID-19 cases worldwide by week, from 1 March 2020 to 31 August 2020. For this 6-month observation period, the linear regression profile (R^2^ = 0.9539) of the spread of COVID-19 appears to indicate that a large expansion of the SARS-CoV-2 virus was underway. Additionally, the COVID-19 spectrum, shown in Figure 1c, has a width of $\left[8\;20\right]\frac{1}{T}$, calculated with a threshold of 5% of the maximum amplitude, with T being the period, i.e., T = 1 week.

2. COVID-19 spread from 1 September 2020 to 28 February 2021

Figure 1d shows the number of COVID-19 cases worldwide by week, from 1 September 2020 to 28 February 2021. For this 6-month observation period, a quadratic regression profile (R^2^ = 0.8122) of the spread of COVID-19 appears to indicate some stability in the expansion of the SARS-CoV-2 virus. The COVID-19 spectrum, shown in Figure 1e, has a width of $\left[12\;16\right]\frac{1}{T}$, i.e., a third of that in Figure 1c, and is therefore in agreement with the hypothesis of stability of the spread of the SARS-CoV-2 virus during this period of time.

3. COVID-19 spread from 1 March 2021 to 31 August 2021

Figure 1f shows the number of COVID-19 cases worldwide by week, from 1 March 2021 to 31 August 2021. For this 6-month observation period, a polynomial regression profile of order 5 (R^2^ = 0.9276) is observed. However, it is important to note that the COVID-19 spectrum, shown in Figure 1g, has a width of [11 17]$\;\frac{1}{T}$ and represents a 50% increase over that in Figure 1e.

## 4. Discussion

### 4.1. COVID-19 Data Reliability

The COVID-19 data reported in this study, i.e., the number of cases and deaths, were extracted daily from the Worldometer platform [30] and were fully consistent with those of the World Health Organization [31]. For obvious reasons, developing countries have not been able to provide their data with the same frequency as developed countries, but it is believed that such a disadvantage does not skew the results of this investigation as much. 

Additionally, at the end of data compilation in August 2021, China was not officially among the top 50 countries (out of 200+) ranked in terms of the total number of COVID-19 cases. Today, almost two and a half years later, in February 2024, China is ranked 92nd, with 503,300 total cases. Given the etiology of COVID-19 [1] and the fact that China is one of the two most populous countries in the world, these statistics may be questioned.

### 4.2. Spread of COVID-19 in G20 Countries

As of September 2021, the analysis of case and death data showed that COVID-19 mainly affects G20 countries. As shown in Table 1, the top 11 countries ranked in terms of the total number of COVID-19 cases are exclusively G20 members, while 80% of the top 20 and 73% of the top 30 are G20 members; this outlook remains essentially the same today, almost two and a half years later, in February 2024.

It seems relevant to note that 9 of the 10 countries (90%) with the most medals at the 2020 Summer Olympics [36] are in the list of top 30 countries ranked by total number of COVID-19 cases (Table 1), with the only exception being China, discussed in the section above. Additionally, 28 of the top 30 countries (93%) in terms of total COVID-19 cases were medalists at the 2020 Summer Olympics. Since developed countries usually top the medal table at the Summer Olympics, this provides further evidence that COVID-19 has primarily affected G20 countries.

### 4.3. Impact of Population Density on the Spread of COVID-19

Table 2 indicates that cities (or municipal areas) with high population density are a powerful activator of the spread of COVID-19. Indeed, the main cities (and municipalities) most affected by COVID-19 generally have a population density greater than several thousand inhabitants. This is probably the most important result of this investigation, as it highlights a potential solution to counter possible future pandemics to come.

The transmission of SARS-CoV-2 through direct person-to-person contact has been recognized since the early stages of the COVID-19 pandemic [8]. Additionally, it has also been observed that the virus can be transmitted through the inhalation of virus-laden aerosols [9,10]. In both cases, because the risk of transmission is higher when people are in close proximity, it is clear that the virus is fully activated in densely populated cities.

Although it seems easier to say than to implement, it might be wise to start thinking about depopulating densely populated cities (and municipalities) in favor of less populated ones. This is becoming a societal problem that developed countries around the world will face sooner or later and therefore needs to be adequately addressed. In fact, densely populated cities may lack quality services, especially in the context of a pandemic. This was particularly true for health services at the start of the COVID-19 pandemic in many countries, leading to a heavy burden of deaths.

### 4.4. What Is Learned from the Spectral Analysis of COVID-19

The observation of the first months of spread of COVID-19, from 1 March 2020 to 31 August 2020, seems to be the most striking, with a strong expansion of the SARS-CoV-2 virus, as indicated by a broad spectrum width in Figure 1c.

The following six months, from 1 September 2020 to 28 February 2021, seem to indicate some stability in the expansion of the SARS-CoV-2 virus, as shown in Figure 1e, with a reduction in the width of the spectrum by 67%. This is mainly a posteriori to the multiple measures that have been taken around the world to counter the spread of COVID-19, namely confinement, the closure of non-essential services, the wearing of masks, distancing of 2 m, etc.

The last six months studied, from 1 March 2021 to 31 August 2021, show a locally periodic signal (Figure 1f), with a 50% increase in the width of the spectrum then being induced (Figure 1g). This probably results from a mixture of opposing and alternating measures, the most notable being confinement and deconfinement. Variants of SARS-COV-2 could also potentially fit into this picture.

### 4.5. The Very Next Pandemic Could Be Closer Than Expected

From the year 1 anno Domini until today (2024), our planet has survived close to twenty pandemics, starting with the Antonine Plague in 165 and ending with COVID-19 in 2019. For the purposes of this discussion, only pandemics that resulted in one million or more deaths per year, regardless of their duration, are reported, as Table 3 illustrates.

**Figure 2:**
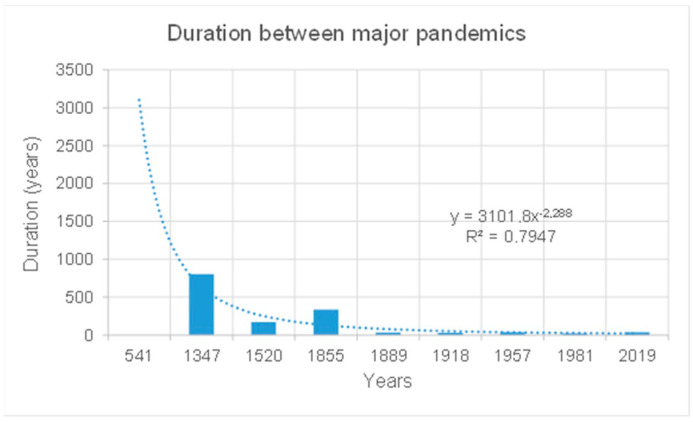
From the year 1 anno Domini until 1855 with the third plague, major pandemics occurred on average every 348 years; since then, they have occurred on average every 33 years, with COVID-19 now underway.

In addition, from the year 1 anno Domini until 1855, the year of the third plague, major pandemics occurred on average every 348 years; since then, they have occurred on average every 33 years [37], as illustrated in Figure 2. In other words, the very next major pandemic could be closer than expected.

### 4.6. Limitations of the Study

This study revisited and analyzed 18 months of data on cases and deaths in the early stages of the COVID-19 pandemic. The spectral analysis highlighted that the very first months of the spread of COVID-19 were the most notable, with a strong expansion of the SARS-CoV-2 virus, while the following six months showed a certain level of stability, mainly due to multiple preventive measures such as changes in national regulations rules to counter the spread. It would have been informative to extend this analysis over a longer period. However, such an investigation would have required a very complex multiparametric model that additionally took into account emerging SARS-CoV-2 variants, the impact of vaccination, etc.; this is well beyond the scope of the current study.

Even if the number of cases and deaths recorded worldwide shows that the pandemic mainly affects G20 countries, its real impact on developing countries remains more or less unknown. Indeed, the latter do not necessarily have effective communication services to provide reliable data at the same frequency as developed countries.

This study shows that cities (or municipal areas) with very high population density are a powerful activator of the spread of COVID-19. This is essentially a quantitative observation. On the other hand, in addition to the high risk of SARS-CoV-2 transmission associated with people in close proximity, the socioeconomic context could also influence the spread of the virus. However, the correlation between the spread of COVID-19 and socioeconomic factors is beyond the scope of the current study.

## 5. Conclusions

Today, phenomenological knowledge, as reported in Table 3 and Figure 2, tends to indicate that the frequency of major pandemics has increased considerably and that the next one could occur in less than 50 years.

The COVID-19 pandemic has taught us that population densities in cities (and municipalities) have a significant impact on the burden of pandemics in terms of cases and then deaths.

Although new knowledge and emerging technologies can provide new vaccines in a relatively short period of time, vaccination remains a primarily curative solution in the context of a pandemic. Indeed, vaccines do not necessary prevent viral infection but mainly aim to minimize its impact on the physiological system.

It is perhaps time to launch new societal projects aimed at relieving congestion in densely populated regions. This seems an adequate solution to minimize the impact of future pandemics.

Even though new knowledge and emerging technologies have considerably improved the human way of life, new challenges arise to optimize and maintain these achievements but also to intelligently prepare for the future. COVID-19 has made us understand that it is time to act both preventatively and curatively.

## Figures and Tables

**Table 1:** Ranking the top 30 countries by total COVID-19 cases as of September 2021. G20 countries, printed in blue, accounted for 81% of total COVID-19 cases and 83% of total COVID-19 deaths.

List of Top 30 Countries Ranked by Total Number of COVID-19 Cases as of September 2021						
Rank	Country	Total Cases	Total Deaths	Population	Area (km^2^)	Population Density
1	USA	80,912,619	983,837	334,252,383	9,834,000	34
2	India	42,962,953	515,063	1,425,775,850	3,287,263	434
3	Brazil	29,033,052	651,988	215,089,085	8,510,000	25
4	France	23,011,998	139,243	65,515,351	551,695	119
5	UK	19,119,181	162,008	68,483,074	243,610	281
6	Russia	16,861,793	355,537	146,039,239	17,100,000	9
7	Germany	15,723,907	124,670	84,232,506	357,592	236
8	Turkey	14,326,217	95,379	85,858,254	783,562	110
9	Italy	12,991,055	155,782	60,312,960	302,073	200
10	Spain	11,100,428	100,431	46,785,101	506,030	92
11	Argentina	8,934,328	126,708	45,890,064	2,780,000	17
12	Iran	7,084,306	137,747	85,792,424	1,648,000	52
13	Netherlands	6,640,403	21,608	18,001,900	41,850	430
14	Colombia	6,070,616	139,037	51,790,765	1,141,748	45
15	Poland	5,734,042	112,535	37,777,204	322,575	117
16	Indonesia	5,723,858	149,918	278,365,371	1,905,000	146
17	Mexico	5,554,392	319,604	131,200,388	1,973,000	66
18	Japan	5,274,596	24,604	125,828,159	377,973	333
19	Ukraine	4,862,459	106,485	43,293,825	603,628	72
20	Vietnam	4,292,564	40,726	98,804,778	331,690	298
21	S. Korea	4,212,652	8796	51,343,064	100,210	512
22	South Africa	3,683,172	99,543	60,560,331	1,220,000	50
23	Israel	3,669,119	10,274	9,326,000	22,145	421
24	Philippines	3,666,672	56,879	112,022,278	300,439	373
25	Czechia	3,624,963	38,911	10,742,247	78,867	136
26	Malaysia	3,595,172	33,173	33,060,108	330,803	100
27	Belgium	3,586,292	30,259	11,674,074	30,688	380
28	Peru	3,524,504	210,995	33,740,598	1,285,215	26
29	Australia	3,344,617	5403	25,995,140	7,688,000	3
30	Portugal	3,322,134	21,182	10,146,927	92,152	110
**Total**		**362,444,064**	**4,978,325**	**3,807,699,448**		

**Table 2:** Summary view of the population density of the cities and municipal areas among the most affected in their respective countries by COVID-19. The ranking of cities (or municipal areas) based on the total number of COVID-19 cases in their respective countries (or states) as of September 2021 is also presented.

Population Densities (PD: Inhabitants per Square Kilometer) of Cities and Municipalities among the Most Affected by COVID-19						
Cities or Municipalities			States		Countries	
Rank	Name	PD	Name	PD	Name	PD
1	Buenos Aires	15,372			Argentina	17
2	Cordoba	2274				
1	Sao Paulo	8149			Brazil	25
5	Rio de Janerio	4836				
1	Montréal-Nord	7623	Québec	6	Canada	4
1	Peel	1283	Ontario	14		
1	Bogota	4100			Colombia	42
2	Prague	2737			Czechia	136
2	London	5598			England	260
1	Cologne	2649			Germany	235
2	Munich	4988				
3	Hanover	2600				
1	Tokyo	6511			Japan	328
2	Osaka	5740				
1	Putrajaya	1387			Malaysia	99
1	Mexico City	6163			Mexico	
1	Utrecht	3705			Netherlands	461
2	Rotterdam	2995				
1	Quezon City	17,666			Philippines	369
2	Cavite	2835				
3	Laguna	1725				
1	Moscow	10,900			Russia	8
2	Saint-Petersburg	3850				
1	Gyeonggi	1335			South Korea	531
2	Seoul	17,000				
3	Busan	4791				
1	Kyiv city	3531			Ukraine	71
1	Los Angeles	3206	California	98	USA	36
2	San Diego	1636				
1	Brooklyn	14,917	New York	166		
2	Queens	8542				
3	Manhattan	28,154				
1	Hanoi	2398			Vietnam	290
2	Ho Chi Minh City	4481				
3	Hi Phong	1358				

**Table 3:** Major pandemics in the Christian era that resulted in at least one million deaths per year. “Year diff” gives the difference in years between the respective beginnings of pandemic N + 1 and pandemic N, N ≥ 1. The “*” in “End (y)” column indicate that these pandemics, namely HIV/AIDS and COVID-19, are not quite over.

List of Some Major Pandemics in the Christian Era						
Pandemic Name	Begin (y)	End (y)	Duration (ys)	Death Toll	Death Toll/y	Yr Diff
Plague of Justinian	541	542	1	40,000,000	40,000,000	---
Black death (Bubonic Plague)	1347	1351	4	200,000,000	50,000,000	806
Smallpox	1520	1520	1	56,000,000	56,000,000	173
The Third Plague	1855	1855	1	12,000,000	12,000,000	335
Russian Flu	1889	1890	1	1,000,000	1,000,000	34
Spanish Flu	1918	1919	1	45,000,000	45,000,000	29
Asian Flu	1957	1958	1	1,100,000	1,100,000	39
HIV/AIDS	1981	2022 *	41	42,100,000	1,026,829	24
COVID-19	2019	2023 *	4	6,961,398	1,740,350	38
